# Aflatoxins as a risk factor for liver cirrhosis: a systematic review and meta-analysis

**DOI:** 10.1186/s40360-020-00420-7

**Published:** 2020-06-01

**Authors:** Abraham Nigussie Mekuria, Michael N. Routledge, Yun Yun Gong, Mekonnen Sisay

**Affiliations:** 1 Department of Pharmacology, School of Pharmacy, Haramaya University, P.O. Box 235, Harar, Ethiopia; 2 School of Medicine, University of Leeds, Leeds, UK; 3 School of Food and Biological Engineering, Jiangsu University, Zhenjiang, Jiangsu Province China; 4 School of Food Science and Nutrition, University of Leeds, Leeds, UK; 5 Department of Pharmacology, School of Pharmacy, Haramaya University, Harar, Ethiopia

**Keywords:** Aflatoxin, mycotoxin, Liver cirrhosis, Chronic liver disease, Meta-analysis

## Abstract

**Background:**

Liver cirrhosis is characterized by fibrosis and nodule formation in the liver, due to a chronic injury, and subsequent alteration of the normal architecture of the liver. Even though there is a huge effort to elucidate the possible etiologic factors of liver cirrhosis, a significant number of cases are cryptogenic, especially in Sub Saharan Africa, where there is a high burden of aflatoxin exposure. Aflatoxins are known to cause hepatocellular carcinoma, which share similar etiologic factors with liver cirrhosis. This study aimed to assess the association between aflatoxin exposure and the risk of liver cirrhosis.

**Methods:**

Relevant studies were identified through systematic searches conducted in Ovid MEDLINE, PubMed and Google Scholar. Also, by searching the references of retrieved articles. The abstracts and full text were screened for eligibility and the risk of bias was assessed for each study using Joanna Briggs Institute (JBI) critical appraisal checklist for observational studies. The extracted data from included studies using Microsoft Excel were exported to Stata software version 15.0 for analyses. The overall pooled estimation of outcomes was calculated using a random-effects model of DerSimonian–Laird method at a 95% confidence level. The heterogeneity of studies was determined using I2 statistics. The presence of publication bias between studies was evaluated using the Begg’s and Egger’s tests and funnel plot. The protocol of this systematic review and meta-analysis was registered in the Prospero database with reference number ID: CRD42019148481.

**Results:**

A total of 5 studies published between the years 2005 and 2018 that met the pre-defined inclusion and exclusion criteria were included. The meta-analysis showed that a significant increase in the risk of liver cirrhosis is associated with aflatoxin exposure (unadjusted pooled odds ratio (OR) = 3.35, 95% CI: 2.74–4.10, *p =* 0.000; I^2^ = 88.3%, *p =* 0.000; adjusted OR = 2.5, 95% CI: 1.84–3.39, *p =* 0.000; I^2^ = 0%, *p =* 0.429).

**Conclusions:**

The present meta-analysis suggests that aflatoxin exposure is associated with a higher risk of liver cirrhosis.

## Background

Cirrhosis is characterized by fibrosis and nodule formation in the liver, secondary to a chronic injury, which leads to alteration of the normal lobular organization of the liver [1, 2]. Cirrhosis is currently the 11th most common cause of death globally and liver cancer is the 16th leading cause of death; when combined, they account for 3.5% of all deaths worldwide [3]. Despite the tremendous amount of progress in our understanding the etiology of liver cirrhosis, many cases are cryptogenic, i.e. cirrhosis of the liver of undetermined etiology [4]. This is true especially in Sub Saharan Africa, where hepatitis B virus (HBV), hepatitis C virus (HCV) and alcohol consumption are involved in 34, 17, and 18% of cases as etiologic factors. However, in 31% of cases, the etiology is unknown, according to a recent global burden of disease report [5].

On the other hand, cirrhosis and hepatocellular carcinoma (HCC) are known to share numerous common etiologic factors, including chronic infection with HBV and/or HCV, heavy alcohol consumption, and non-alcoholic steatohepatitis/non-alcoholic fatty liver disease [5, 6]. An additional etiologic factor for HCC is exposure to aflatoxins (AFs) through the consumption of AF contaminated foods [7]. In this regard, Sub Saharan Africa is an area with a diet particularly high in AFs [8–10].

Emerging evidence has indicated that AF exposure may be involved in the pathogenesis of liver cirrhosis [11, 12]. Though there is no clear causation between AF and liver cirrhosis, the mutational activity of AF has been considered to be the main factor of AF-induced HCC [13]. As both AF exposure and liver cirrhosis are the main risk factors of HCC, it remains unclear whether AF also contributes to the earlier stage of HCC progression, i.e., liver cirrhosis. The objective of this systematic review was to analyze existing research to test the hypothesis that AFs cause liver cirrhosis by meta-analysis approach.

## Methods

### Study protocol

The Preferred Reporting Items for Systematic Review and Meta-analysis (PRISMA) guideline was used to report the finding of this review [14]. This systematic review and meta-analysis was conducted by following the PRISMA Protocol [15]. The completed checklist has been provided as supplementary material (Additional file 1: Table S1). The study protocol is registered on PROSPERO with reference number ID: CRD42019148481.

### Inclusion/exclusion criteria

During the screening and assessment of full texts for eligibility, there were predefined inclusion-exclusion criteria to arrive at the final included papers. Observational studies (Case-control or cohort studies) addressing AF exposure as a risk factor for liver cirrhosis were included. There were no restrictions on publication year, but only studies that were written in English were considered for inclusion. Studies having irretrievable full texts (after requesting full texts from the corresponding authors via email and/or Research Gate account) or studies with unrelated or insufficient outcome measures or studies with outcomes of interest that are missing or vague were excluded.

### Data sources and search strategy

We performed an electronic literature search until December 31st, 2019, on Ovid MEDLINE and PubMed: using the following keywords and indexing terms: ‘aflatoxin’, ‘liver cirrhosis’, and ‘chronic liver disease’. Advanced Google Scholar search was also conducted to identify other relevant published and unpublished works including dissertations, institutional repositories, and organizational manuals, among others. Boolean operators (AND, OR) and truncation were used when appropriate to increase the number of relevant findings. Additionally, we searched (back-traced) reference lists from retrieved articles to identify further relevant studies.

### Screening and eligibility of studies

The documents identified from different electronic sources were exported to ENDNOTE reference software version 7.8 (Thomson Reuters, Stamford, CT, USA) with compatible formats. Duplicate documents were removed with the help of ENDNOTE and manually. Each of the documents retrieved was assessed by the authors independently for eligibility by reading the title, abstract using the preset inclusion and exclusion criteria. This process was followed by retrieval and assessment of the full texts of the relevant citations. Any disagreement was solved by discussion.

### Data extraction

Data extraction format prepared in Microsoft Excel was developed to extract data from each included study. The authors independently extracted the data related to study characteristics and outcome measures: including authors, publication year, study design and populations, study location, study period, diagnostic method, number of cases and controls, the age and sex of study subjects, method of AF exposure assessment (dietary intake of AF contaminated foods and biomarkers of AF exposure [*249ser TP53* mutation, AF-albumin adduct, AF-N7-guanine adducts excreted in urine]), risk ratios (RRs)/odds ratios (ORs) and their 95% CI with or without adjustment for confounding factors, and variables adjusted for analysis, if any.

### Critical appraisal of studies

To maintain methodological validity, before the inclusion of the eligible articles they were assessed by two independent reviewers using the Joanna Briggs Institute (JBI) critical appraisal checklist for case-control and cohort studies [16]. The assessment tool consisted of 10 questions about the quality of the study for which articles received values representing the extent to which they met the following criteria: Yes, No, Unclear and Not applicable. This critical appraisal was conducted to assess the internal (systematic error) and external (generalizability) validity of studies and to reduce the risk of biases. The mean score of the two authors was taken for final decision and studies with a score greater than or equal to five out of 10 were considered low risk and included in the study.

### Outcome measurements

Our primary outcome of interest in this meta-analysis was the association between AF exposure and the risk of liver cirrhosis. Subgroup analyses based on information on the study design, geographic location and method of exposure assessment were performed.

### Data processing and statistical analysis

The extracted data were exported from Excel to STATA 15.0 software *for analyses of outcome measures and sub-grouping. Considering the variation in true effect sizes across the population, Der-Simonian-Laird’s random-effects model was applied for the analysis at 95% confidence level. The significance of heterogeneity of the studies was assessed using I*^*2*^*statistics* based on Cochran’s Q test, I^2^ returns and the percent variation across studies. The presence of publication bias was evaluated using the Begg’s and Mazumdar’s correlation and Egger’s regression tests and presented with funnel plots [17, 18]. A statistical test with a *p*-value of less than 0.05 was deemed to be significant.

## Results

### Search result

**Fig. 1:**
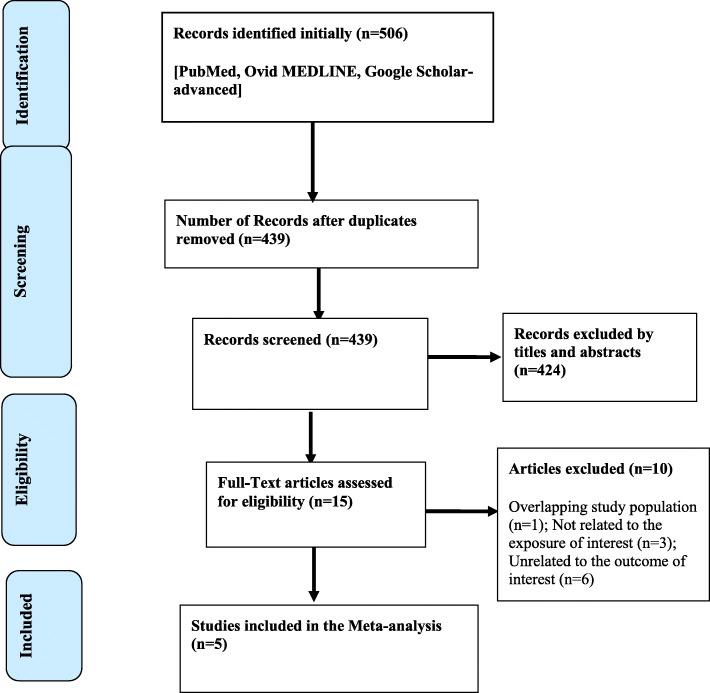
PRISMA flow chart describing the selection process

As shown in Fig. 1, the search identified 506 studies, of which 67 studies were found to be duplicates. From the 439 remaining records, 424 were excluded after reading their titles and abstracts. Full texts of 15 records were read to assess their eligibility. Of these, 10 records were further excluded because they did not satisfy the inclusion criteria. The remaining 5 studies [12, 19–22] were included in this systematic review and meta-analysis.

### Study characteristics

Among the five studies that met the inclusion criteria, four of them were case-control studies and one study was a nested case-control study. They were conducted in Gambia [19, 20], Taiwan [12], India [21], and China [22] and involved 941 cases and 2, 281 controls. The included studies were published between 2005 and 2018. As shown in Table 1, the included studies employed AF-albumin adduct level [12, 21, 22], 249ser TP53 mutation [19–21] and groundnut consumption [19, 20] as methods of AF exposure assessment in liver cirrhosis patients. As depicted in Table 1, three of the included studies reported unadjusted and adjusted ORs and two studies [21, 22] did not report the adjusted odds ratio. Most studies were adjusted for factors such as age, gender, cigarette smoking, and alcohol drinking; two studies [19, 20] were also adjusted for recruitment site and date, socioeconomic status, HBV, and HCV status.

**Table 1:** Characteristics of studies included for systematic review and meta-analysis

Author & year	Country	Study Design & population	Study period	No of cases (% of Males)	No ofcontrols (% of Males)	Method of AF exposure assessment	Unadjusted OR (95%CI)	Adjusted OR (95%CI)	Adjusted variables	Result of critical appraisal
Wang, 2018 [22]	China	Hospital based case-control	2008–2012	384 (75.3)	851 (75.7)	AF-albumin adduct	7.74 (5.51–10.87)	^a^	^a^	Low risk
Chu, 2017 [12]	Taiwan	Community-based nested case-control	1991–2004	232 (^a^)	577 (^a^)	AF-albumin adduct	2.29 (1.44–3.64)	2.45 (1.51–3.98)	Age, gender, cigarette smoking, alcohol drinking, ALT	Low risk
Anitha, 2014 [21]	India	Hospital based case-control	2009–2010	130 (^a^)	108 (^a^)	AF-albumin adduct	3.59 (1.56–8.23)	^a^	^a^	Low risk
						249ser TP53 mutation	3.46 (0.72–16.7)			
Kuniholm, 2008 [20]	Gambia	Hospital based case-control	1997–2001	97 (62.9)	397 (71)	249ser TP53 mutation	3.9 (1.8–8.4)	3.8 (1.5–9.6)	Age, gender, recruitment site & date, socioeconomic status, alcohol, tobacco, HBV, HCV	Low risk
						Ground nut intake	2.6 (1.2–5.8)	2.8 (1.1–7.7)		
Kirk, 2005 [19]	Gambia	Hospital based case-control	1997–2001	98 (65.3)	348 (69.8)	249ser TP53 mutation	5.06 (2.28–11.22)	4.83 (1.71–13.7)	Age, gender, recruitment date & site, ethnicity, alcohol, socioeconomic status, HBV & HCV status	Low risk
						Ground nut intake	0.8546 (0.53–1.37)	1.79 (1.04–3.08)		

*Abbreviations*: *AF* Aflatoxin, *ALT* Alanine transaminase, *HBV* Hepatitis B virus, *HCV* Hepatitis C virus

^a^Not reported

### AF exposure and risk of liver cirrhosis

**Fig. 2:**
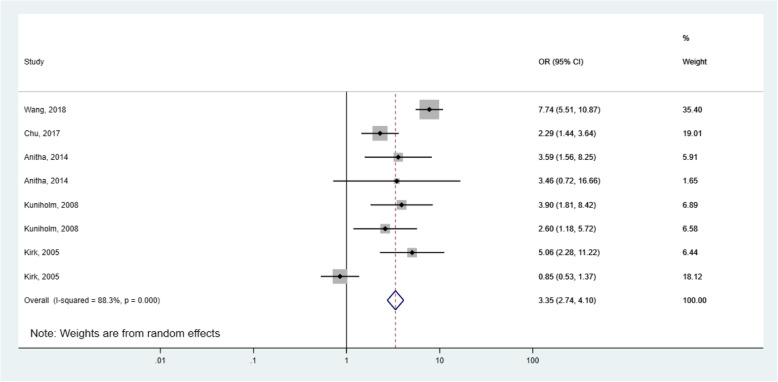
Forest plot of aflatoxin exposure and risk of liver cirrhosis using unadjusted odds ratios

After pooling, the five studies that reported the unadjusted OR suggested a significantly higher risk of liver cirrhosis associated with AF exposure (OR = 3.35, 95% CI: 2.74–4.10, *p =* 0.000). However, high evidence of heterogeneity (I^2^ = 88.3%, *p =* 0.000) was observed in the pooled estimate (Fig. 2).

**Fig. 3:**
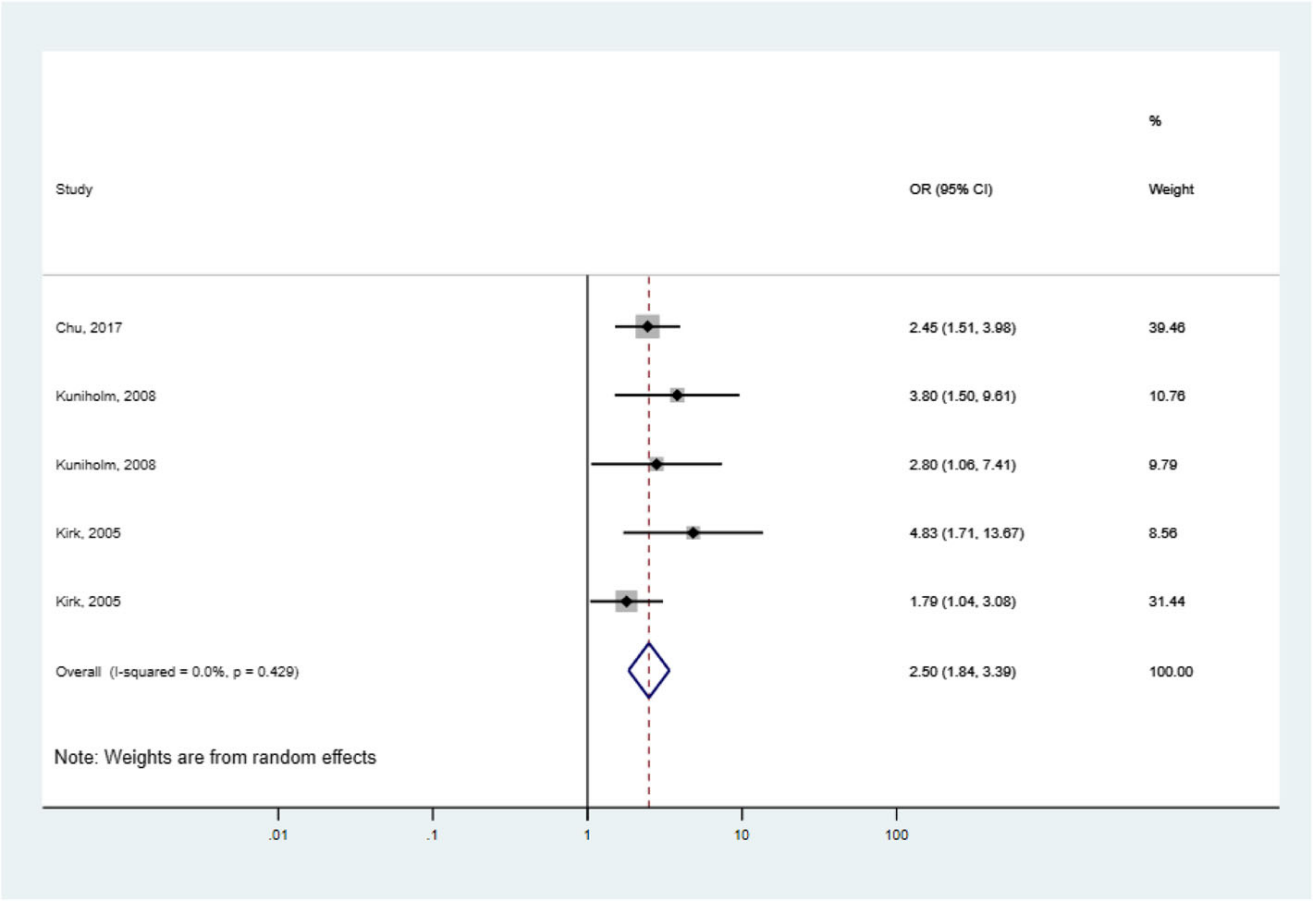
Forest plot of aflatoxin exposure and risk of liver cirrhosis using adjusted odds ratios

On the other hand, after pooling of the adjusted OR estimates of individual studies, AF exposure was still associated with a higher risk of liver cirrhosis (OR = 2.5, 95% CI: 1.84–3.39, *p =* 0.000) and no evidence of heterogeneity (I^2^ = 0%, *p =* 0.429) was found in the pooled estimate and subgroup analysis (Fig. 3).

### Subgroup analyses

As shown in Table 2, subgroup analyses by study design, AF exposure assessment method and geographical region of study populations were performed to identify the sources of heterogeneity in the unadjusted OR estimates of individual studies. In the subgroup analysis by study design, the pooled estimate of case-control was 3.67 (95% CI: 2.93–4.59, *p =* 0.000; I^2^ = 89.4%, *p =* 0.000). In the subgroup analysis by AF exposure assessment method, the pooled estimate revealed that there was a significant association between AF-albumin adduct and liver cirrhosis [4.89 (95% CI: 3.77–6.35, *p =* 0.000; I^2^ = 88.8%, *p =* 0.000)], as well as between 249ser TP53 mutation and liver cirrhosis [4.30 (95% CI: 2.55–7.26, *p =* 0.000; I^2^ = 0.00%, *p =* 0.863)] though no statistically significant association was observed between groundnut consumption and liver cirrhosis [1.15 (95% CI: 0.76–1.72, *p =* 0.51; I^2^ = 82.4%, *p =* 0.017)].

**Table 2:** Subgroup analyses of AF exposure and risk of liver cirrhosis using unadjusted ORs

Subgroup	Studies, N	OR (95% CI)	*p* value	Tests for heterogeneity		
				Q	*p*	I^2^
All studies	8	3.35 (2.74,4.10)	0.000	59.58	0.000	88.3%
Study design						
Case-control studies	7	3.67 (2.93,4.59)	0.000	56.38	0.000	89.4%
Nested case-control studies	1	2.29 (1.44,3.64)	0.000	0.00	–	–
Method of AF exposure assessment						
Serum AF-albumin adduct level	3	4.89 (3.77, 6.35)	0.000	17.83	0.000	88.8%
249ser TP53mutation	3	4.3 (2.55,7.26)	0.000	0.30	0.863	0.0%
Groundnut consumption	2	1.15 (0.76,1.72)	0.51	5.68	0.017	82.4%
Geographic location						
Asia	4	4.85 (3.75,6.26)	0.000	18.01	0.000	83.3%
Africa	4	1.84 (1.32,2.55)	0.000	20.75	0.000	85.5%

*AF* Aflatoxin, *OR* Odds ratio, *CI* Confidence interval

In the subgroup analysis performed by geographical region, the corresponding pooled OR for Asia was 4.85 (95% CI: 3.75–6.26, *p =* 0.000; I^2^ = 83.3%, *p =* 0.000), and that of the African region was 1.84 (95% CI: 1.32–2.55, *p =* 0.000; I^2^ = 85.5%, *p =* 0.000) (Table 2).

### Publication bias

**Fig. 4:**
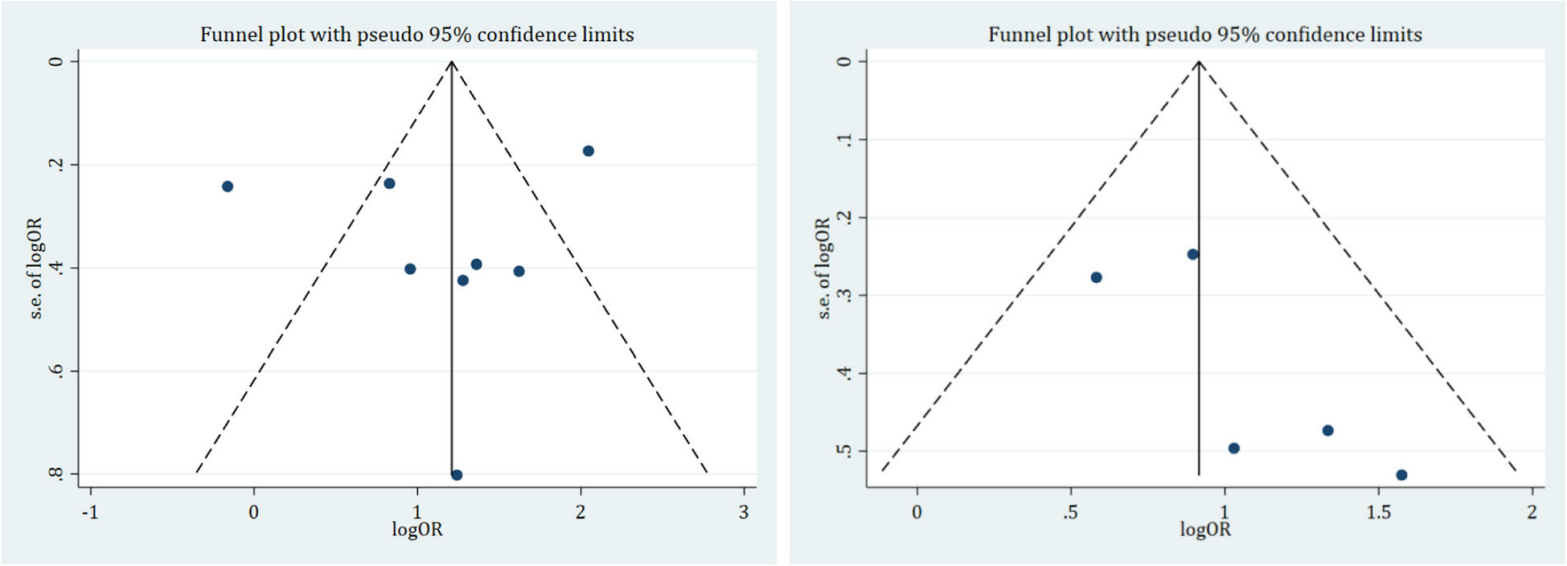
Funnel plot depicting publication bias (unadjusted and adjusted odds ratios)

The presence of publication bias was depicted using funnel plots of log OR and standard error of it and supplemented with statistical tests: Egger’s regression test (*p =* 0.683 for unadjusted ORs and *p =* 0.122 for adjusted ORs) and Begg’s and Mazumdar’s correlation test (continuity corrected) (*p =* 1.00 for unadjusted OR and *p =* 0.22 for adjusted OR) (Fig. 4). The finding indicated that there is no evidence of statistically significant publication bias among the included studies.

## Discussion

This study is the first systematic review and meta-analysis to investigate the relationship between exposure to AF and the risk of liver cirrhosis. The results of the present study showed a significant association between AF exposure and the risk of liver cirrhosis. Despite the heterogeneity presented for most studies, those studies that performed the adjusted tests were able to demonstrate homogeneity in the comparisons. Subgroup analysis was conducted to reduce the degree of heterogeneity among studies. The random effect model has also been applied considering the variability of the effect size.

A likely explanation of this association is not yet identified, though consumption of AF-contaminated foods and feeds were reported to cause diverse degrees of liver injury comprising development of fatty cysts, fibrosis, and cirrhosis among humans and animals [23–27]. However, several lines of evidence support oxidative stress as a key factor in AF induced initiation and progression of liver cirrhosis [28–31].

The toxic effects of AFB1 against the liver and other organs are closely related to its metabolic activation into the free radical AFB1-exo-8,9-epoxide (AFBO) by cytochrome P450 (CYP450) enzymes [32] and associated formation of reactive oxygen species (ROS) including hydroxyl radical (HO^.^), per hydroxyl radical (HOO^−^) and superoxide anion [29, 33]. This can result in oxidative stress owing to an imbalance between limited antioxidant defenses and the excessive formation of ROS, resulting in the damage of biological molecules including lipids, proteins, and DNA in cellular systems [34, 35]. In support of this hypothesis, several studies have demonstrated the potential for antioxidants to lower the risk of hepatotoxicity caused by exposure to the AF [29, 36–39].

Moreover, many studies have reported the pivotal role of oxidative stress induced by AF in eliciting programmed cell death or apoptosis through mitochondrial signaling pathways [25, 40–42]. ROS induced mitochondrial damage is known to cause uncoupling of mitochondrial oxidative phosphorylation and the associated reduction in mitochondrial membrane potential following AFB1 administration in vivo and in vitro [25, 33, 35]. Consequently, mitochondrial alterations cause activation of cytochrome C that modulates Bcl2/Bax gene expression and activate caspase 9 and caspase 3, which results in cell death [41].

## Conclusions

The current meta-analysis indicates that AF exposure is significantly associated with liver cirrhosis. However, large sample studies using standardized unbiased AF exposure assessment methods and well-matched controls are required to support this association further.

## Supplementary information

**Additional file 1: Table S1.** Completed PRISMA checklist. The checklist highlights the important components addressed while conducting systematic review and meta-analysis from observational studies.

## Data Availability

All data generated or analyzed in this study are included in this article.

## Abbreviations

HCC: Hepatocellular carcinoma
HBV: Hepatitis B virus
HCV: Hepatitis C virus
AF: Aflatoxin
JBI: Joanna Briggs Institute
OR: Odds ratio
CI: Confidence interval
ROS: Reactive Oxygen Species
